# Integrating NRG1 and OXT Polymorphisms, Maternal Psychological Vulnerability, and Obstetric–Neonatal Outcomes: A Genotype–Phenotype Pilot Study

**DOI:** 10.3390/jcm15166286

**Published:** 2026-08-14

**Authors:** Ioana Denisa Socol, Simona Sorina Farcaș, Flavius George Socol, Bogdan-Ionel Dumitriu, Daniela Iacob, Nicoleta Ioana Andreescu

**Affiliations:** 1 Doctoral School, “Victor Babeș” University of Medicine and Pharmacy, 300041 Timișoara, Romania; ioana.socol@umft.ro (I.D.S.); bogdan.dumitriu@umft.ro (B.-I.D.); 2 Genomic Medicine Centre, “Victor Babeș” University of Medicine and Pharmacy, 300041 Timișoara, Romania; andreescu.nicoleta@umft.ro; 3 Discipline of Neonatology, “Victor Babeș” University of Medicine and Pharmacy, 300041 Timișoara, Romania; daniela.iacob@umft.ro; 4 Discipline of Genetics, Department of Microscopic Morphology, Faculty of Medicine, “Victor Babeș” University of Medicine and Pharmacy, 300041 Timișoara, Romania; 5 1st Clinic of Obstetrics and Gynecology, “Pius Brînzeu” County Clinical Emergency Hospital, 300723 Timișoara, Romania; george.socol@umft.ro

**Keywords:** pregnancy complications, neuregulin-1, oxytocin, polymorphism, single nucleotide, anxiety disorders

## Abstract

**Background and Objectives:** Maternal psychological vulnerability during pregnancy is associated with adverse obstetric and neonatal outcomes, but the contribution of candidate genetic markers remains insufficiently defined. This exploratory pilot case–control study examined NRG1 rs35753505, NRG1 rs3924999, and OXT rs2740210 together with validated psychological screening and obstetric–neonatal phenotyping in Romanian primiparous women. **Methods:** Eighty-two primiparous women were enrolled between February 2024 and February 2025 at a tertiary maternity centre in Timișoara. After genotyping quality control, 79 women with complete data for all three SNPs (50 cases and 29 controls) formed the analytic sample. Fifty women with at least one obstetric complication were compared with 29 women with uncomplicated pregnancies. Participants completed the EPDS, GAD-7, PSS-10, and CD-RISC-25 at 20–28 gestational weeks. Genotyping was performed using TaqMan^®^ allelic discrimination assays, with Hardy–Weinberg equilibrium assessment and exploratory carrier, allele-dosage, correlation, ANOVA, and logistic-regression analyses. **Results:** The study lot had higher EPDS, GAD-7, and PSS-10 scores and lower CD-RISC-25 scores than controls (all *p* < 0.001). The three SNPs were in Hardy–Weinberg equilibrium and did not show statistically significant single-locus case–control differences. NRG1 rs3924999 A-carriers showed the strongest exploratory association pattern, including lower neonatal birth weight and higher psychological vulnerability scores. Across exploratory genetic risk score (GRS) tertiles, birth weight, Apgar-5, perceived stress, and resilience differed significantly; however, the formal multiplicative GRS × PSS-10 model for low birth weight was not significant. Stratified correlations suggested that the PSS-10-birth weight relationship was stronger in the high-GRS tertile. **Conclusions:** NRG1 rs3924999 and the composite NRG1/OXT score may help define future replication hypotheses, but larger genotype-stratified cohorts are required before clinical risk stratification can be proposed. The composite score is an investigator-weighted, internally derived exploratory index rather than a validated genetic risk model, and the formal gene–environment interaction test was null, so the stratified correlations are descriptive only. The findings should be interpreted as hypothesis-generating.

## 1. Introduction

Gestation is a period of accelerated biological and psychological change during which the maternal nervous, endocrine, and immune systems must adapt rapidly to the metabolic demands of the developing fetus [1]. This adaptive process unfolds against a background of fluctuating social, economic, and personal stressors, and evidence from perinatal cohorts and meta-analyses indicates that perceived stress, anxiety, depressive symptoms, and reduced resilience may be associated with preterm birth, gestational hypertension, intrauterine growth restriction (IUGR), and low birth weight (LBW) [2,3,4,5], including in our own Romanian primiparous cohort [6]. However, these associations vary between individuals and should not be interpreted as deterministic.

Nevertheless, not all women exposed to equivalent levels of psychological distress develop complications, and the molecular substrate underlying inter-individual variability in stress reactivity remains incompletely understood [7], as summarised in our recent systematic review and meta-analysis of genetic determinants of stress reactivity in pregnancy [8]. Candidate-gene research in pregnancy has most frequently focused on HPA-axis and stress-regulation pathways, but these systems explain only part of the wider neurobiological network that may shape maternal–fetal stress reactivity [9,10].

Two complementary gene systems deserve greater attention in this context. Neuregulin-1 (NRG1) is a trophic factor expressed throughout the developing nervous system that orchestrates neuronal migration, myelination, synaptic plasticity, and placental trophoblast invasion [11]. Common variants in NRG1 have been linked to schizophrenia, affective disorders, and altered stress reactivity in adults, and NRG1 is strongly expressed in the human placenta [11,12]. The single-nucleotide polymorphisms (SNPs) rs35753505 (SNP8NRG243177) and rs3924999 lie in regulatory and coding regions of NRG1 and have been proposed as candidate modulators of neuropsychiatric vulnerability [12].

The oxytocin gene (OXT) encodes the neuropeptide oxytocin, which is central to parturition, lactation, maternal bonding, and attenuation of the stress response [13]. Variants in OXT and its receptor have been associated with postpartum depression, trait anxiety, and sensitivity to social support during pregnancy [13]. The intronic SNP rs2740210 has been linked to variations in maternal behaviour and stress reactivity, and therefore represents a plausible candidate locus for perinatal outcome research [13].

Despite these mechanistic leads, empirical studies combining psychological phenotyping with NRG1 and OXT genotyping in pregnant women and linking both to objective obstetric and neonatal endpoints remain limited. Most available studies examine single markers or broader stress-pathway mechanisms; therefore, analyses of the NRG1/OXT axis should be presented as exploratory and interpreted cautiously, especially in modest sample sizes [9,10,11,12,13,14].

The available evidence is, however, neither uniform nor uncontested. For NRG1, the initial haplotype findings have proved difficult to reproduce: meta-analytic synthesis indicated that the pooled association with schizophrenia was driven largely by early, small studies and was accompanied by substantial between-study heterogeneity [15], and subsequent appraisal found the evidence to weaken as larger samples accumulated [16]. Evidence for oxytocin-pathway variants is similarly mixed, with genotype effects on maternal mood and caregiving emerging chiefly in interaction with early experience rather than as main effects, and in directions that vary with the outcome measure used [17], and with oxytocin-receptor effects on observed parenting proving small and inconsistently replicated [18]. More broadly, the candidate-gene and candidate gene-by-environment literature from which these hypotheses derive has been criticised for low statistical power, flexible analytical strategies, and publication bias [19], with large well-powered analyses providing no support for historically prominent candidate loci in depression [20] and with early positive genetic associations attenuating or disappearing on replication as a general rule [21]. Perinatal studies are additionally heterogeneous in the timing of psychological assessment, the definition of obstetric endpoints, and the handling of population stratification, which limits direct comparability. These considerations do not remove the biological rationale for examining NRG1 and OXT in pregnancy, but they establish that a single-centre pilot analysis of these loci must be positioned as hypothesis-generating rather than confirmatory.

In summary, three strands of evidence converge on the present research question. First, antenatal psychological vulnerability is a reproducible correlate of adverse obstetric and neonatal outcomes, yet its effects differ markedly between individuals exposed to comparable levels of distress. Second, NRG1 and OXT are biologically credible modulators of that inter-individual variability, acting on neurodevelopmental, placental, and stress-regulatory pathways relevant to the same endpoints. Third, the existing genetic evidence for both loci is limited, heterogeneous, and derived predominantly from non-obstetric populations, so its relevance to pregnancy outcomes is untested rather than established. What is missing is a study in which validated psychological phenotypes, NRG1 and OXT genotypes, and objectively documented obstetric–neonatal endpoints are measured in the same primiparous women. The present pilot study was designed to address that gap.

The present pilot study therefore focused on the less-explored NRG1/OXT axis. We examined whether (i) the distributions of NRG1 rs35753505, NRG1 rs3924999, and OXT rs2740210 differed between primiparous women with and without obstetric complications; (ii) carrier- and dosage-based exploratory analyses suggested relationships with antenatal psychological distress, resilience, and neonatal outcomes; and (iii) the association between maternal perceived stress and birth weight differed across exploratory GRS tertiles. The objective was to generate replication hypotheses rather than to establish a clinically actionable genetic risk model. We prespecified one primary and two secondary hypotheses. The primary hypothesis was that primiparous women who subsequently developed an obstetric complication would show greater antenatal psychological vulnerability—higher EPDS, GAD-7, and PSS-10 scores and lower CD-RISC-25 scores—than women with uncomplicated pregnancies. The secondary hypotheses were that (i) carriage of the prespecified NRG1 rs35753505 T, NRG1 rs3924999 A, and OXT rs2740210 A alleles, considered individually and combined in an exploratory composite score, would be associated with higher psychological vulnerability scores and with less favourable obstetric and neonatal outcomes, including lower birth weight, earlier gestational age at delivery, and more frequent NICU admission; and (ii) the inverse association between maternal perceived stress and neonatal birth weight would be stronger in women with a higher composite score, a pattern consistent with the differential-susceptibility framework. The secondary hypotheses were treated as exploratory throughout, and no power calculation was performed for the genetic analyses.

## 2. Materials and Methods

### 2.1. Study Design & Setting

This investigation was designed as a prospective, observational case–control pilot study. Recruitment took place between 1 February 2024 and 1 February 2025 at the 1st Clinic of Obstetrics and Gynecology of the “Pius Brinzeu” County Emergency Clinical Hospital in Timisoara, Romania, a tertiary, university-affiliated level III maternity centre that serves a large region of south-western Romania (counties Timis, Arad, Caras-Severin, Hunedoara, and Mehedinti). This geographic catchment provides adequate socio-economic heterogeneity while preserving relative ethnic homogeneity, a useful feature for exploratory candidate-gene analyses.

During the same outpatient visit at 20–28 gestational weeks, 82 enrolled women consented to provide an additional peripheral blood sample for NRG1 and OXT genotyping. One sample failed DNA quality control and was excluded, leaving 81 evaluable participants. The OXT rs2740210 assay additionally failed in one case and one control; therefore, 79 women (50 cases and 29 controls) with complete data for all three SNPs formed the analytic sample. Eligibility required primiparity, age 18–45 years, a stable partnership, uninterrupted employment for at least 24 months before conception, a minimum of secondary education, a viable singleton pregnancy, and the absence of obstetric complications or hospitalisations at enrolment. Exclusion criteria covered pre-existing psychiatric diagnoses, current psychotropic treatment, substance use, documented COVID-19 hospitalisation within three years before conception, assisted reproductive technology, prior adverse pregnancy outcomes, chronic corticosteroid or immunomodulatory therapy, active infection, domestic violence, homelessness, and major placental or fetal anomalies. All women provided written informed consent. The study was approved by the Ethics Committee of the “Pius Brinzeu” Clinical Emergency Hospital in Timisoara (approval no. 17/31 January 2024) and complied with the 1964 Declaration of Helsinki and its later amendments. These criteria were applied to limit non-genetic variance in a pilot sample. Primiparity was required because parity modifies the risk of hypertensive disease, preterm birth, and fetal growth restriction. A stable partnership, uninterrupted employment, and a minimum of secondary education served as proxies for a stable psychosocial and socio-economic environment, limiting variance attributable to acute financial insecurity, unemployment, housing instability, and limited health literacy. Pre-existing psychiatric diagnoses and current psychotropic treatment were excluded because the screening instruments are calibrated for untreated populations, and assisted reproduction, prior adverse pregnancy outcomes, and chronic corticosteroid or immunomodulatory therapy were excluded as established competing causes of the studied endpoints. The resulting cohort is partnered, employed, predominantly urban, and of relatively homogeneous ethnic background, and is therefore not representative of unselected obstetric populations.

### 2.2. Study Groups and Outcome Definitions

Participants were classified into two groups on the basis of the final obstetric outcome documented at delivery. The study lot (*n* = 50, coded G1–G52 with two cases excluded for incomplete genotyping) consisted of primiparous women who developed at least one of the following a priori defined complications: preterm birth (PTB; delivery before 37 completed gestational weeks), gestational hypertension (GHT; new-onset hypertension after 20 weeks without proteinuria), intrauterine growth restriction (IUGR; ultrasound-estimated fetal weight below the 10th percentile for gestational age), or low birth weight (LBW; neonatal weight below 2500 g). The control lot (*n* = 29, coded M1–M30, one control excluded for incomplete OXT genotyping) included primiparous women with uneventful pregnancies, term delivery, and appropriately grown neonates.

Because participants in the study lot could present with more than one complication, subgroup analyses were performed for each individual obstetric endpoint, and a composite adverse obstetric outcome was defined as the occurrence of at least one of PTB, GHT, IUGR, or LBW. The composite endpoint was used because the number of events available for each individual complication (35 preterm births, 22 with IUGR, 21 low-birth-weight neonates, and 18 with gestational hypertension) was insufficient for separate genotype comparisons, and because the four conditions share upstream mechanisms of impaired trophoblast invasion and uteroplacental perfusion. The four endpoints nevertheless differ in aetiology and gestational timing, and each component was therefore also analysed separately across score tertiles. Neonatal variables abstracted from delivery records included gestational age at birth, birth weight in grams, Apgar score at 1 and 5 min, and admission to the neonatal intensive care unit (NICU). Umbilical cord blood gas (pH and base excess) was also recorded. Sampling was performed from the umbilical vein rather than the umbilical artery, in accordance with the standing protocol of the delivery unit; venous sampling was applied uniformly to all deliveries during the recruitment period and is technically more reliable in the small-calibre cords of growth-restricted and preterm neonates. Umbilical arterial values reflect fetal acid–base status more accurately, whereas venous values reflect the combined maternal–placental contribution and underestimate fetal acidaemia [22,23]. Cord blood gas variables were used descriptively and were not entered into any inferential model. Each participant was assigned a unique anonymised identifier used throughout laboratory and statistical processing.

### 2.3. Psychological Assessment

Psychological phenotyping used four validated Romanian-language instruments administered during a single outpatient visit between 20 and 28 gestational weeks. The Edinburgh Postnatal Depression Scale (EPDS; 10 items, range 0–30, cut-off ≥ 13) screened depressive symptoms [14]; the Generalised Anxiety Disorder-7 scale (GAD-7; range 0–21, cut-off ≥ 10) assessed anxiety [24]; the Perceived Stress Scale-10 (PSS-10; range 0–40, cut-off ≥ 20) quantified stress appraisal; and the Connor–Davidson Resilience Scale-25 (CD-RISC-25; range 0–100, with lower scores indicating lower adaptive capacity) captured psychological resilience.

Questionnaires were completed in a quiet room under the supervision of trained research staff, who remained available to clarify items on request while encouraging independent responses. All four instruments have been validated in Romanian obstetric samples and display satisfactory internal consistency. Cut-offs were applied in accordance with the most commonly used thresholds in the perinatal literature, thereby ensuring comparability with international data [6]. All four questionnaires were completed at the single 20–28-week visit, before any of the studied complications had been diagnosed. Absence of obstetric complications and of pregnancy-related hospitalisation at enrolment was an inclusion criterion, and group allocation was determined retrospectively from the obstetric outcome recorded at delivery. Neither the participants nor the research staff supervising the assessment were aware of the eventual group assignment at the time of testing, and the instruments were administered identically in both groups.

### 2.4. Genotyping Procedure

Peripheral venous blood (4 mL, EDTA) was collected immediately after the psychological assessment. Genomic DNA was extracted using the MagCore Nucleic Acid Extraction Kit (RBC Bioscience, New Taipei City, Taiwan), in accordance with the manufacturer’s instructions. DNA concentration and purity were then determined using an Epoch Microplate Spectrophotometer (Agilent BioTek, Santa Clara, CA, USA) to confirm sample quality before further analysis. All sample processing and genotyping were performed at the Center of Genomic Medicine, “Victor Babeș” University of Medicine and Pharmacy, Timisoara, Romania. Three SNPs were genotyped using TaqMan^®^ allelic discrimination assays (Applied Biosystems, Thermo Fisher Scientific, Waltham, MA, USA) on a real-time PCR platform: NRG1 rs35753505 (alleles C/T), NRG1 rs3924999 (alleles A/G), and OXT rs2740210 (alleles A/C). Amplification reactions were performed on the LightCycler 480 Real-Time PCR System (Roche Diagnostics, Basel, Switzerland), with genotype discrimination conducted using Gene Scanning software, version 1.5.1. All reactions were executed in a 96-well plate format, maintaining a standardized volume of 20 µL per well. A minimum of 10% of samples were re-genotyped blindly for quality control, and concordance exceeded 99%.

Genotypes were first tabulated descriptively as homozygous reference, heterozygous, and homozygous alternate categories within study lot and controls. Because several genotype-specific cells were small, the primary inferential analyses used exploratory carrier coding (carrier vs. non-carrier of the prespecified allele: rs35753505-T, rs3924999-A, and rs2740210-A) and additive allele-dosage coding (0, 1, or 2 allele copies). In the dosage models, allele dosage denotes the number of copies (0, 1, or 2) of the prespecified allele carried by each participant; accordingly, “T-dosage” for rs35753505 and “A-dosage” for rs3924999 and rs2740210 refer to the count of T or A alleles, respectively, at the corresponding locus, rather than fully powered homozygote-vs.-heterozygote subgroup comparisons within each study group. A three-SNP exploratory composite allele-dosage index, abbreviated GRS for brevity, was calculated as GRS = 1.2 × NRG1_rs35753505_T-dosage + 1.0 × NRG1_rs3924999_A-dosage + 1.3 × OXT_rs2740210_A-dosage and then categorised into tertiles. These coefficients were prespecified for exploratory scaling and should not be interpreted as evidence that OXT rs2740210 has greater biological importance than the NRG1 variants; the score was used only for hypothesis generation. The score was not derived from genome-wide data, was not weighted by externally estimated effect sizes, and has not been validated internally or externally. The weights were assigned a priori by the investigators on grounds of biological plausibility rather than estimated from data; the three variants were selected from the literature that generated the study hypothesis; and the tertile boundaries were derived within the present sample rather than at prespecified absolute cut-points. No cross-validation, bootstrap shrinkage, or split-sample validation was performed. Hardy–Weinberg equilibrium was evaluated separately in the study lot, controls, and the pooled sample.

### 2.5. Statistical Analysis

All analyses were performed in Python 3.12 using the pandas, scipy, and statsmodels libraries, with a two-sided alpha set at 0.05. Continuous variables are presented as mean ± standard deviation (SD); categorical variables as absolute frequencies and percentages. Case–control comparisons of continuous variables used Welch’s *t*-test, and categorical variables were compared with chi-square or Fisher’s exact tests where appropriate. Hardy–Weinberg equilibrium (HWE) was assessed for each SNP using the classical chi-square goodness-of-fit test with one degree of freedom. Genotype distributions were reported descriptively, while inferential comparisons relied on carrier and allele-dosage models because the study was not powered for stable three-genotype subgroup analyses. Standardised mean differences (SMD) were calculated for all baseline variables: Cohen’s d using the pooled standard deviation for continuous variables, the difference in proportions divided by the pooled standard deviation of the proportions for binary variables, and Cramér’s V for genotype distributions. Absolute values below 0.10 were regarded as negligible and values above 0.40 as indicating imbalance warranting consideration as a confounder [25].

Bivariate relationships between psychological scores, the exploratory GRS, and neonatal outcomes were evaluated using Pearson correlations for continuous outcomes and point-biserial correlations for dichotomous outcomes. For comparisons across GRS tertiles, one-way ANOVA was used for continuous outcomes and Pearson chi-square tests for categorical outcomes. Multivariable logistic regression assessed the independent contribution of the GRS and each psychological score to NICU admission, adjusting for maternal age. A GRS × PSS-10 interaction term was added to a logistic model for LBW within cases, and stratified Pearson correlations between PSS-10 and birth weight by GRS tertile were used only as an exploratory visual and descriptive assessment. The interaction model was restricted to the study lot because low birth weight was confined to that group by definition, controls having been defined by term delivery with appropriately grown neonates; including controls would have added observations with no outcome variance. The restricted model comprised 50 women with 21 low-birth-weight events, approximately four events per estimated parameter, and a truncated range for both PSS-10 and the composite score. The model was additionally refitted post hoc in the full sample (*n* = 79) with group membership retained as a covariate (interaction OR = 1.04, 95% CI 0.95–1.14, *p* = 0.401). Because of the pilot sample size, all analyses are interpreted as hypothesis-generating and no causal or clinical predictive claims are made. Comparisons were performed across four psychological instruments, three SNPs under two coding schemes, a composite score, and multiple obstetric and neonatal endpoints. All reported *p*-values are nominal and were not adjusted for multiplicity. The Benjamini–Hochberg procedure for control of the false discovery rate at 5% was applied post hoc to the ten comparisons across score tertiles [26], and the resulting q-values are reported with those comparisons.

## 3. Results

Table 1 summarises the baseline characteristics and genotype distributions. The study lot and controls were broadly comparable for age, gestational age at enrolment, residence, and marital status. Standardised mean differences are reported alongside the significance tests so that the magnitude of imbalance can be judged independently of the sample size. Age (SMD = 0.08), gestational age at enrolment (SMD = 0.14), marital status (SMD = −0.18), and urban residence (SMD = 0.24) showed negligible to small imbalance. BMI showed small-to-moderate imbalance (SMD = 0.34) despite a non-significant *p*-value of 0.095. Higher education showed the largest imbalance of any baseline variable (66.0% of cases versus 41.4% of controls; SMD = 0.51), a moderate difference that the borderline *p*-value of 0.054 understates at this sample size, and educational attainment should therefore be regarded as a potential confounder rather than as a balanced covariate. Genotype distributions were closely comparable between groups (Cramér’s V = 0.10–0.14), and none of the three differed significantly at the nominal *p* < 0.05 threshold. All results use the same complete-case sample: 50 cases and 29 controls (79 women with successful genotyping for all three SNPs).

Table 2 shows that the study lot had higher EPDS, GAD-7, and PSS-10 scores and lower CD-RISC-25 scores than controls (all *p* < 0.001). The proportions above the conventional clinical cut-offs followed the same direction, supporting a clear psychological vulnerability pattern in the complication group without implying causality.

Table 3 reports genotyping quality-control results. All three SNPs satisfied Hardy–Weinberg equilibrium in the pooled sample and in each subgroup at *p* > 0.05. These findings support the internal consistency of the genotyping data, although the small sample size limits the interpretation of minor allele-frequency differences. Hardy–Weinberg equilibrium and allele frequencies were assessed in the complete-case sample of 79 women (50 cases, 29 controls) with successful genotyping for all three SNPs; three women were excluded for incomplete genotyping (one failed all three assays; two failed OXT rs2740210).

Table 4 presents exploratory carrier-based and allele-level comparisons. None of the three single-locus comparisons reached statistical significance, and the confidence intervals were wide. Therefore, the observed directions of effect should be treated only as preliminary signals requiring replication rather than as evidence of risk or protection for any individual allele.

Table 5 reports bivariate correlations between psychological scores and obstetric–neonatal outcomes. Higher EPDS, GAD-7, and PSS-10 scores were generally associated with lower birth weight and shorter gestation, while CD-RISC-25 showed positive associations with neonatal outcomes. These correlations support an association between antenatal psychological vulnerability and neonatal indicators, but they do not establish causality or a specific biological pathway.

Table 6 presents the multivariable logistic regression model for NICU admission. The clearest result of this analysis is negative with respect to the genetic hypothesis: the exploratory composite score made no independent contribution to the prediction of NICU admission (OR = 0.79, 95% CI 0.48–1.28, *p* = 0.335), with a confidence interval comfortably spanning unity and a point estimate directionally opposite to that hypothesised. By contrast, all three psychological measures remained independently associated with NICU admission after mutual adjustment and adjustment for maternal age: each one-point increase in GAD-7 was associated with a 22% increase in the odds of admission (OR = 1.22, 95% CI 1.06–1.40, *p* = 0.006), each one-point increase in PSS-10 with a 13% increase (OR = 1.13, 95% CI 1.02–1.26, *p* = 0.020), and each one-point increase in CD-RISC-25 with a 5.5% decrease (OR = 0.95, 95% CI 0.90–1.00, *p* = 0.039). Within this model the antenatal psychological phenotype rather than the genotype carried the predictive signal. GAD-7, PSS-10, and CD-RISC-25 retained statistical significance after mutual adjustment, whereas the exploratory GRS and maternal age did not. The model fit was acceptable for a pilot analysis, but the estimates should be interpreted cautiously because of the modest number of complete observations and events.

**Figure 1:**
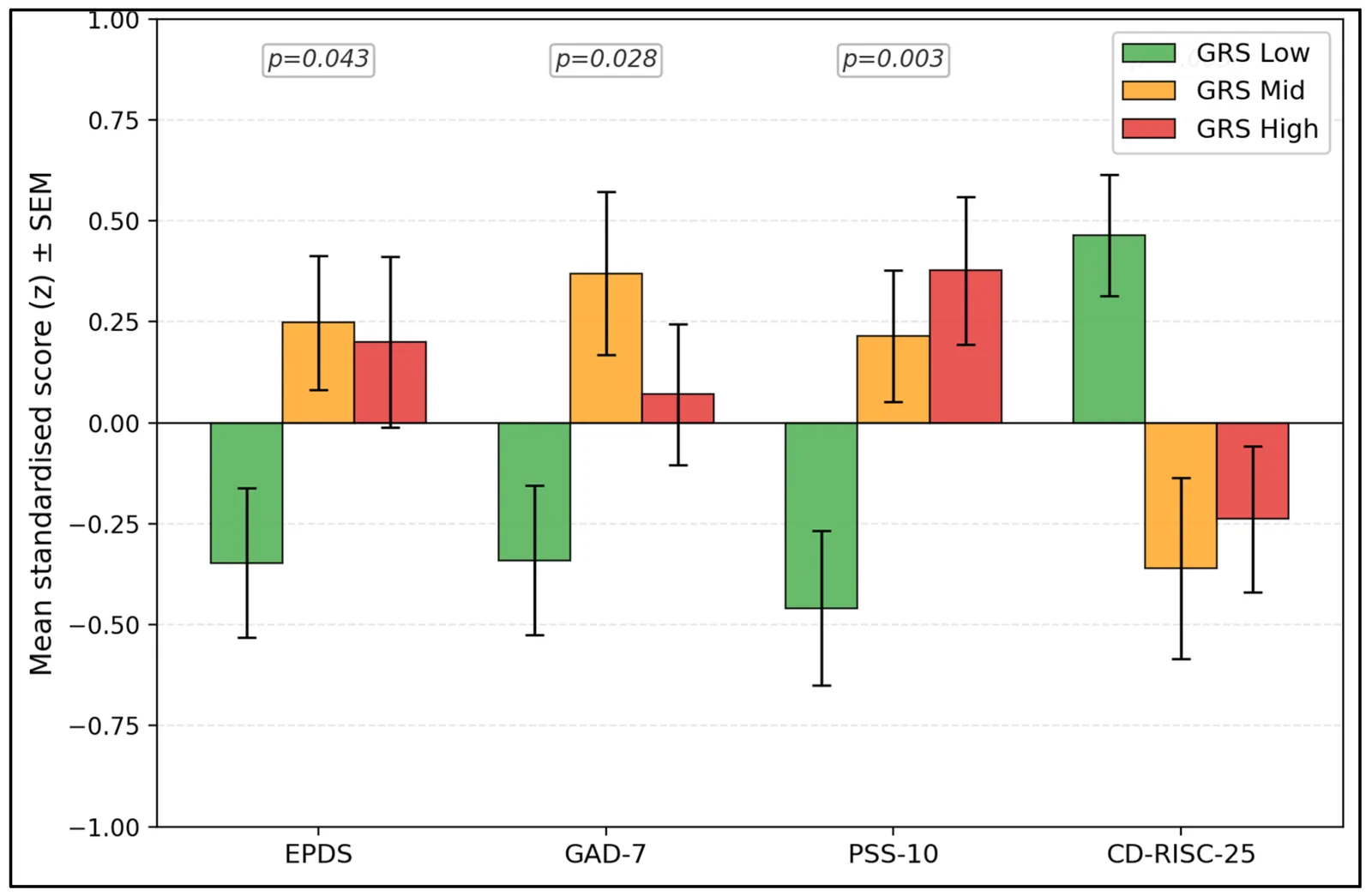
Exploratory comparison of standardised antenatal psychological scores by GRS tertile. Bars represent z-standardised means (± SEM) for EPDS, GAD-7, PSS-10, and CD-RISC-25; *p*-values are from one-way ANOVA across tertiles.

Figure 1 shows that psychological scores differed across exploratory GRS tertiles, with higher vulnerability scores and lower resilience generally observed in the mid/high tertiles. These findings are descriptive and should be interpreted as hypothesis-generating, particularly because genotype-specific subgroups were small.

**Figure 2:**
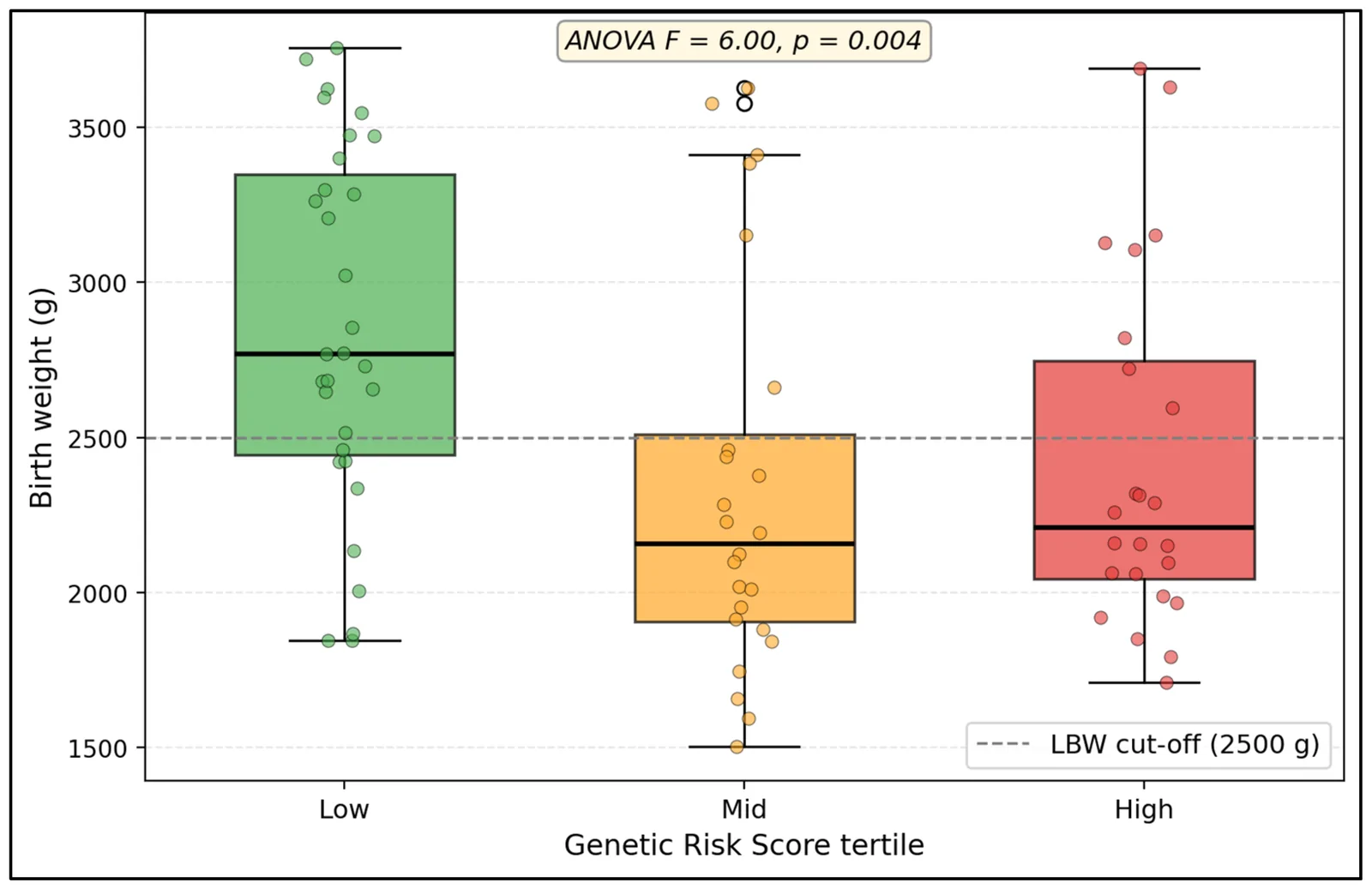
Exploratory birth-weight distribution across GRS tertiles. Box-and-whisker plots include individual data points; the horizontal dashed line marks the 2500 g low-birth-weight threshold. Green, orange, and red denote the low, mid, and high GRS tertiles, respectively.

Figure 2 shows lower mean birth weight in the mid and high GRS tertiles than in the low tertile (ANOVA *p* = 0.004). Because the pattern is not strictly linear, the result is best described as a tertile-based difference rather than definitive proof of a dose–response relationship.

**Figure 3:**
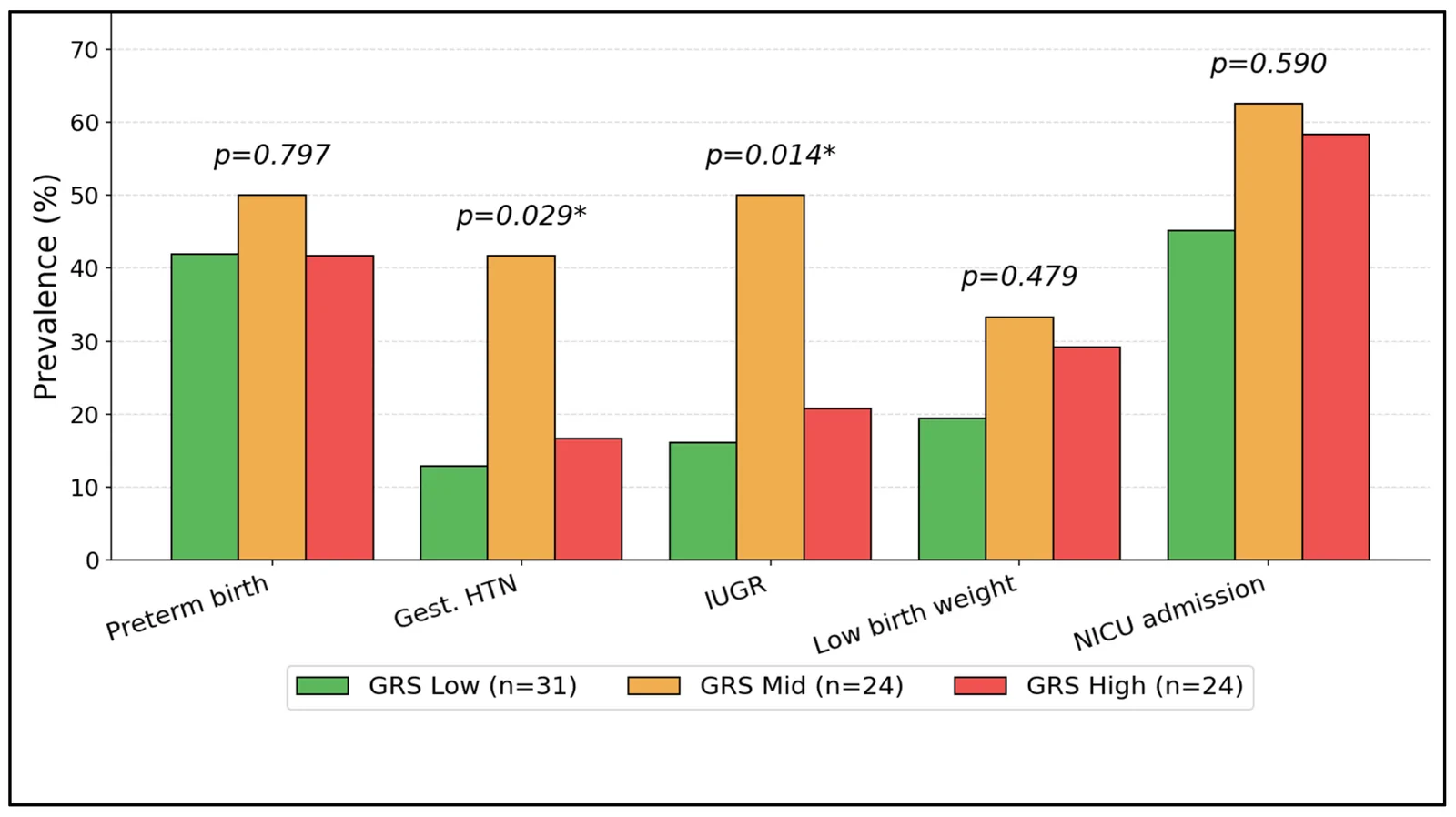
Exploratory prevalence of obstetric and neonatal complications across GRS tertiles in the complete-case sample (*n* = 79). Grouped bars show the percentage of women in each tertile developing each complication; *p*-values are from Pearson chi-square tests. Asterisks denote statistically significant differences between tertiles (* *p* < 0.05).

Figure 3 suggests tertile-level differences for IUGR and gestational hypertension, with the highest proportions in the middle tertile. Other outcomes did not differ significantly. The non-monotonic pattern argues against overinterpreting the GRS as a simple linear genetic-risk gradient.

**Figure 4:**
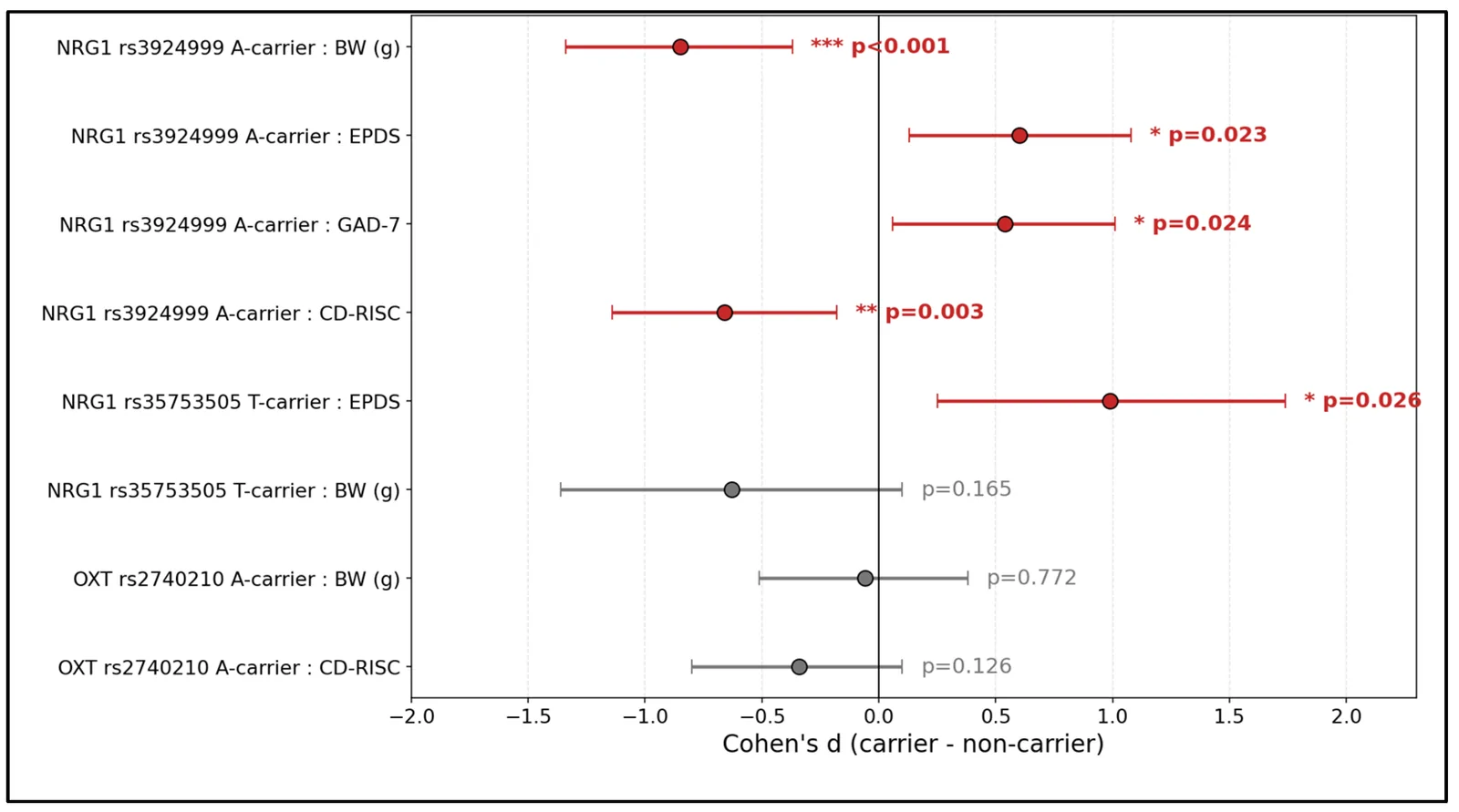
Exploratory standardised carrier effects (Cohen’s d with 95% CI) for selected psychological and neonatal outcomes in the complete-case sample (*n* = 79). Red markers indicate *p* < 0.05; grey markers indicate non-significant findings. Asterisks denote the level of statistical significance (* *p* < 0.05; ** *p* < 0.01; *** *p* < 0.001).

Figure 4 indicates that NRG1 rs3924999 A-carriers showed the strongest exploratory carrier-level signal, including lower birth weight and higher psychological vulnerability scores. However, these analyses compare allele carriers with non-carriers rather than fully separating homozygous and heterozygous genotypes; therefore, the findings should be considered preliminary.

Table 7 formally tests the multiplicative GRS × PSS-10 interaction for LBW within the study lot. The interaction term was not statistically significant (OR = 1.03; *p* = 0.633), and the overall model was underpowered. Consequently, the later stratified plots and correlations should be described as exploratory effect-modification signals rather than definitive evidence of a gene–environment interaction.

Table 8 provides stratified correlations between psychological scores and birth weight by GRS tertile. Correlations were weakest in the low tertile and strongest in the high tertile, especially for PSS-10 and GAD-7. This pattern is compatible with possible effect modification, but it remains descriptive because the formal interaction model in Table 7 was not significant. The three tertile-specific coefficients were never formally compared with one another; a difference between correlations computed in separate strata is not itself a test of interaction, and the only such test performed in this study (Table 7) was null. The apparent gradient across tertiles is compatible with sampling variation in subgroups of 24–31 women and must not be read as evidence of gene–environment interaction.

Table 9 summarises psychological and obstetric–neonatal outcomes across exploratory GRS tertiles. Birth weight, Apgar-5, psychological scores, IUGR, and gestational hypertension differed statistically across tertiles, while preterm birth and LBW did not. The categorical comparisons must be interpreted with particular caution because they rest on very small event counts. The IUGR difference (*p* = 0.014) is based on 5, 12, and 5 events across the low, mid, and high tertiles respectively, and the gestational hypertension difference (*p* = 0.029) on 4, 10, and 4 events; several cells therefore contain fewer than five observations, which destabilises the chi-square approximation and leaves each tertile-specific proportion surrounded by wide uncertainty. Both associations are also non-monotonic, peaking in the middle tertile and falling again in the high tertile, which is not the pattern expected if the composite score indexed a graded genetic liability. After Benjamini–Hochberg correction across the ten comparisons in this table both remained below the 5% false-discovery threshold (q = 0.028 and q = 0.041 respectively), and under the same correction the differences in birth weight, PSS-10, and CD-RISC-25 (all q = 0.013), Apgar at 5 min (q = 0.028), and GAD-7 (q = 0.041) also remained significant, whereas the EPDS difference did not (q = 0.054). Correction for multiplicity does not, however, remedy the underlying sparsity: reallocating a small number of participants between tertiles would abolish either categorical association. Because some patterns were non-monotonic, these results should guide replication hypotheses rather than be presented as a definitive dose–response model.

**Figure 5:**
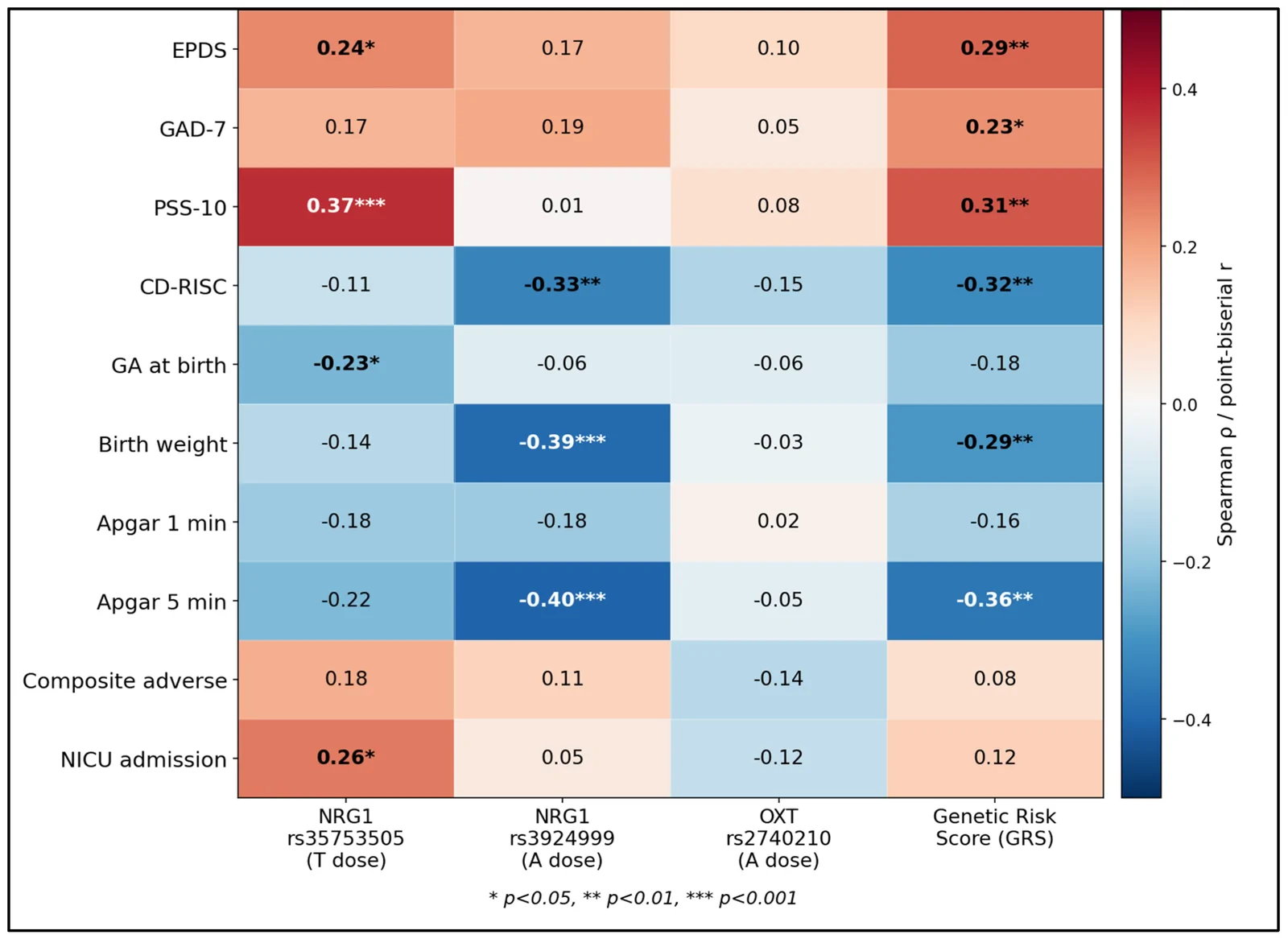
Exploratory correlation matrix linking SNP dosage and the composite GRS with psychological and obstetric–neonatal variables in the complete-case sample (*n* = 79). Cells show Spearman ρ or point-biserial r.

Figure 5 suggests that NRG1 rs3924999 was most consistently related to birth weight, Apgar-5, and resilience, whereas NRG1 rs35753505 T-dosage was more closely related to perceived stress, EPDS, and NICU admission. OXT rs2740210 showed weaker individual correlations.

**Figure 6:**
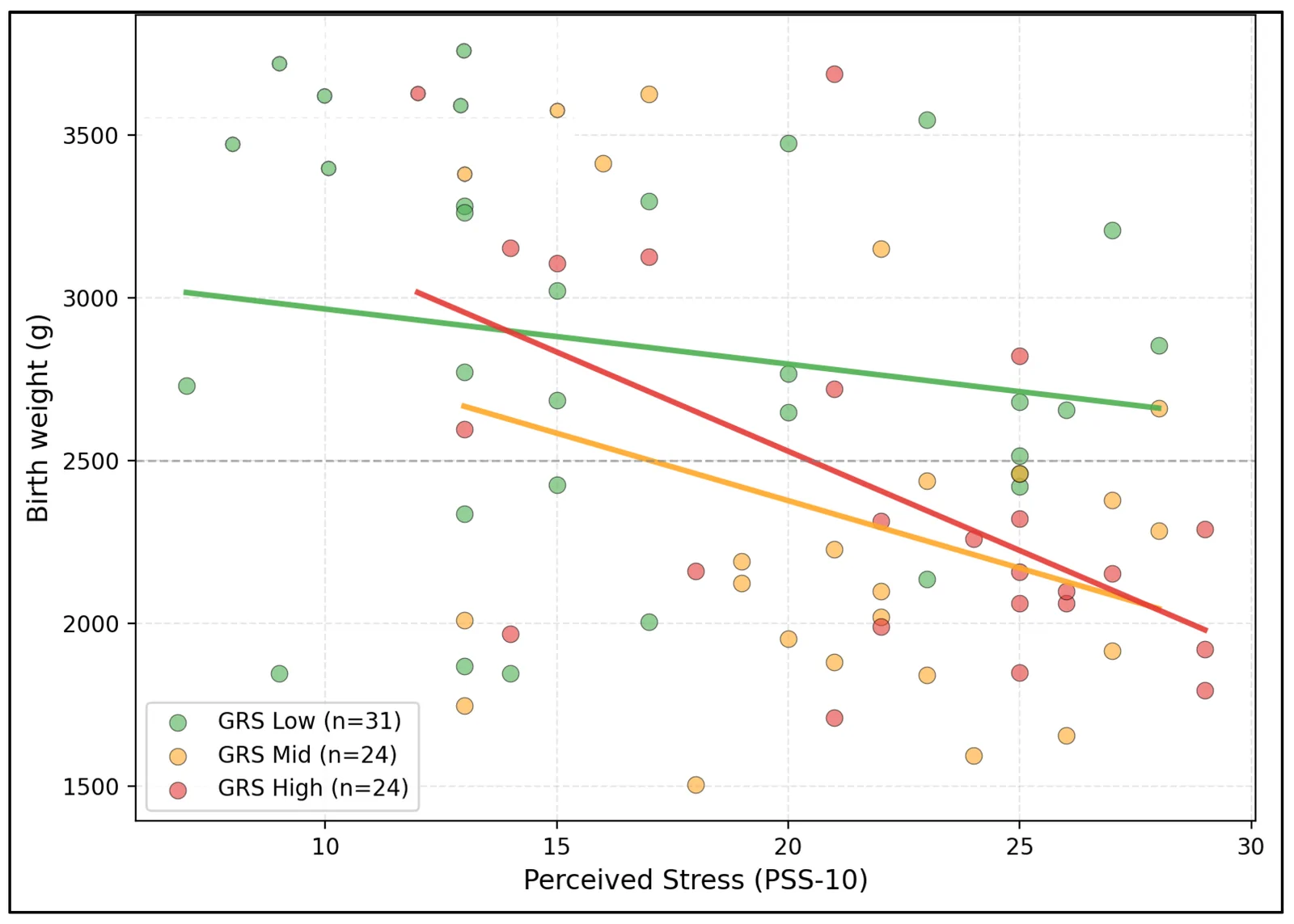
Exploratory stratified association between PSS-10 and birth weight by GRS tertile. The scatter plot shows tertile-specific regression lines; correlation coefficients and *p*-values are reported in Table 8. Green, orange, and red denote the low, mid, and high GRS tertiles, respectively; all three regression lines are solid, and the dashed grey lines are grid lines only.

Figure 6 shows a stronger negative PSS-10–birth weight correlation in the high-GRS tertile than in the low-GRS tertile. This visual pattern supports a hypothesis of possible effect modification, but it should be interpreted alongside the non-significant multiplicative interaction model in Table 7 and requires confirmation in a larger cohort. The regression lines shown are descriptive summaries fitted within tertiles and were not statistically compared.

## 4. Discussion

### 4.1. Analysis of Findings

The psychological phenotype of primiparous women who later developed complications was clearly different from that of controls, with higher EPDS, GAD-7, and PSS-10 scores and lower CD-RISC-25 scores. These findings are consistent with international literature linking antenatal psychological distress to adverse birth outcomes [27,28]. By contrast, the genetic findings were more exploratory: single-SNP case–control comparisons were not significant, while the composite GRS and NRG1 rs3924999 carrier analyses generated signals that require replication in larger cohorts.

The asymmetry between these two sets of findings is the principal message of the study. The psychological differences were large, internally consistent across four independently validated instruments, concordant in direction with the correlation and regression analyses, and highly significant (all *p* < 0.001) despite the modest sample, and they accord with a substantial prior literature [2,3,4,5,27,28]. The genetic findings are of a different evidential quality: no single-locus case–control comparison approached significance, the composite score contributed nothing independently to the NICU model, the formal interaction test was null, and the tertile-level differences that were observed were non-monotonic and rested on small cell counts. A sample of 79 women provides very limited power for genetic association analysis; at the allele frequencies observed here the design has well under 20% power to detect an odds ratio of 1.5, which is at or above the upper end of what is realistic for common variants. Unlike genome-wide polygenic instruments, whose construction, calibration, and predictive limits have been characterised in detail [29,30], the present composite has no external calibration of any kind. The genetic analyses are therefore better described as having been unable to test their hypotheses than as having tested and rejected them, and the reproducible finding of this study concerns maternal psychological phenotype rather than genotype.

The stratified correlation pattern in Figure 6 and Table 8 is compatible with, but does not prove, a differential-susceptibility framework. The differential-susceptibility model holds that individuals differ in their sensitivity to environmental influence rather than simply in their vulnerability to adversity [31,32], and genotype-dependent associations between antenatal mood and offspring outcomes have been reported for other stress-related loci [33,34]. The present data nevertheless do not support such an inference. The only formal test of effect modification performed in this study was the multiplicative GRS × PSS-10 interaction term in Table 7, and it was not significant (OR = 1.03, 95% CI 0.92–1.15, *p* = 0.633; model likelihood-ratio *p* = 0.568), a result unchanged when the model was refitted in the full sample (*p* = 0.401). The stratified correlations in Table 8 and the tertile-specific regression lines in Figure 6 are descriptive summaries of subgroups and not a test of interaction: correlations computed within separate strata provide no evidence that those correlations differ from one another, the differences between them were never formally evaluated, and the apparent gradient is compatible with sampling variation in subgroups of 24–31 women. This study therefore provides no statistical evidence of a gene–environment interaction between the NRG1/OXT composite score and perceived stress. The association between perceived stress and birth weight was strongest in the high-GRS tertile, yet the formal multiplicative interaction model was not significant. Accordingly, the data should be used to formulate future genotype-stratified hypotheses rather than to infer that the maternal psychological environment acts preferentially in genetically vulnerable women.

At the molecular level, the exploratory signal for NRG1 rs3924999 is biologically plausible because neuregulin-1 signalling has been implicated in neural development and placental processes [35,36]. Variation in NRG1 has also been linked to altered gene expression and neurobiological function in the brain [37]. In this cohort, A-carriers were associated with lower birth weight and less favourable psychological scores, but the carrier model does not distinguish heterozygous from homozygous effects with sufficient power. The result should therefore be viewed as a candidate association requiring external validation rather than as a confirmed mechanistic link.

The OXT rs2740210 polymorphism did not show a significant single-locus association in this pilot dataset. Its inclusion in the exploratory GRS was based on biological plausibility related to oxytocin signalling, maternal bonding, parturition, and stress attenuation [38]. However, the present data do not support assigning a firm independent effect to this marker, and the GRS coefficient for OXT should not be interpreted as evidence of stronger documented impact.

Balanced interpretation also requires engagement with the studies that have not supported these loci. For NRG1, the original association signal has proved difficult to reproduce: meta-analysis indicated that the pooled effect was largely attributable to early small studies with pronounced heterogeneity [15], later appraisal found the evidence to weaken as sample sizes increased [16], and the broader NRG1 literature has been described as showing inconsistent genotype–phenotype relationships across independent samples [37]. For OXT the picture is comparably mixed: oxytocin-pathway genotypes have predicted maternal mood and caregiving quality only in conjunction with early experience, and in directions varying with the measure used [17]; oxytocin-receptor effects on observed parenting are small and inconsistently replicated [18]; and the rs2740210 association with postpartum depressive symptoms was itself reported as an interaction with early-life adversity rather than as a main effect [38]. The candidate-gene and candidate gene-by-environment paradigm from which the present design derives has been criticised on the grounds that most positive reports came from underpowered studies with flexible analytical strategies [19], that large-sample analyses provide no support for historically prominent candidate loci in depression [20], that early positive associations commonly attenuate on replication [21], and that such analyses frequently fail to control for confounding of the environmental exposure, which can by itself generate spurious interaction terms [39]. Our own null single-locus results and null interaction test are consistent with this body of work, and the nominally significant tertile-level differences are therefore not presented as support for the NRG1/OXT hypothesis.

The lack of an independent GRS effect in the NICU logistic regression also supports cautious interpretation. Psychological scores retained stronger associations with NICU admission than the composite genetic score. This may reflect limited power, confounding, or partial overlap between psychological and genetic measures, and it reinforces the need for larger studies before genotyping can be considered clinically useful in this context.

### 4.2. Study Limitations

This study has several limitations. First, the analytic sample of 79 women (50 cases, 29 controls) is small for genetic association analysis and precludes stable estimation of genotype-specific effects, especially for homozygous versus heterozygous comparisons within cases or controls. The carrier and dosage models were therefore chosen for feasibility and should be interpreted as exploratory. With 50 cases and 29 controls and the allele frequencies observed here, the study had approximately 15–20% power to detect an odds ratio of 1.5 at a single locus, so the absence of single-locus significance carries essentially no evidential weight. Second, the observational design and single mid-gestational psychological assessment do not allow causal inference or evaluation of distress trajectories. A single assessment between 20 and 28 weeks cannot capture change in symptoms across gestation, cannot distinguish transient from persistent distress, and provides no information about the first or third trimesters. Although all instruments were completed before any complication had been diagnosed and while every participant was formally complication-free, reverse or bidirectional causation cannot be excluded: women with an incipient but undiagnosed complication may already have experienced subclinical symptoms, non-specific somatic discomfort, or anxiety prompted by equivocal earlier findings, and such early awareness could have raised distress scores in precisely the group that subsequently developed complications. Third, the genotyping panel was limited to three candidate SNPs in NRG1 and OXT and did not include other plausible genetic or epigenetic pathways, such as FKBP5, glucocorticoid-receptor methylation, oxytocin-receptor methylation, or placental methylation of cortisol-signalling and steroidogenic genes [33,34,40,41,42]. Fourth, the GRS coefficients were not derived from independent external effect-size estimates; they were used only as exploratory scaling coefficients, and the OXT coefficient should not be interpreted as assigning greater biological importance to OXT than to the NRG1 variants. The composite is exposed to overfitting from several directions simultaneously: the three variants were selected from the same literature that generated the hypothesis, the weights were assigned by the investigators rather than estimated from external data, and the tertile boundaries were derived within this sample, so any apparent performance of the score is optimistic by an unquantified amount. No internal validation, cross-validation, or shrinkage was feasible at this sample size, and the score has not been evaluated in any independent dataset; it should not be described or used as a genetic risk score in the established sense of that term. Fifth, education showed a moderate between-group imbalance (66.0% versus 41.4%; SMD = 0.51) that the borderline *p*-value of 0.054 understates, and educational attainment is plausibly associated both with the reporting of psychological symptoms and with obstetric risk; the sample was too small to adjust reliably for education alongside the other covariates, so residual confounding by socio-economic position cannot be excluded. Several further potential confounders were not quantified at all, including paternal age, paternal health, and paternal psychiatric history; maternal nutrition and dietary quality; ambient air pollution and occupational exposures; physical activity and sleep; and the availability of partner, family, and wider social support, the last of which is both a strong predictor of perceived stress and a plausible modifier of any gene–environment relationship. Sixth, no formal correction for multiple comparisons was applied to the primary presentation, and the number of tests performed makes chance findings likely; the post hoc false-discovery-rate results should be regarded as the appropriate frame for the tertile-level comparisons. Seventh, umbilical cord blood gas was sampled from the umbilical vein rather than the umbilical artery; venous values reflect the combined maternal–placental contribution, underestimate fetal acidaemia, and are a weaker index of intrapartum fetal status than arterial values, which is why these variables were used descriptively and excluded from all inferential models. Eighth, the composite adverse obstetric outcome combines four conditions with distinct aetiologies and differing gestational timing, which may mask endpoint-specific or opposing associations. Ninth, recruitment took place at a single tertiary centre in south-western Romania under deliberately restrictive eligibility criteria, producing a comparatively advantaged, partnered, employed, predominantly urban primiparous sample of relatively homogeneous ethnic background; the findings may not generalise to unselected obstetric populations, to multiparous women, or to other ancestries, and neither genomic control nor ancestry-informative markers were used to exclude residual population stratification. Finally, the multiplicative GRS × PSS-10 model was underpowered and non-significant, so the stratified correlation pattern should be treated as a replication hypothesis. Taken together, these limitations mean that the genetic component of this study should be regarded as a feasibility and hypothesis-generating exercise rather than as a test of association, and that no clinical inference should be drawn from the composite score.

## 5. Conclusions

This pilot case–control study suggests that antenatal psychological vulnerability is associated with adverse obstetric–neonatal indicators and that NRG1/OXT candidate markers may provide exploratory signals for future research. No individual SNP showed a statistically significant case–control association, and the strongest genetic findings involved carrier/dosage models and GRS tertiles rather than fully powered genotype-specific comparisons. NRG1 rs3924999 A-carriers and the composite GRS should therefore be viewed as hypothesis-generating markers, not as validated predictors. The stronger PSS-10–birth weight correlation in the high-GRS tertile is compatible with possible effect modification, but the formal interaction model was non-significant. Because that formal test was the only inferential assessment of effect modification performed, the stronger tertile-specific correlation is a descriptive observation and does not constitute evidence of a gene–environment interaction. The robust finding of this study is the psychological one; the genetic findings, including the exploratory composite score, are unvalidated, are not clinically actionable, and must not be used for risk stratification in any form. Larger longitudinal cohorts with complete genotype-stratified analyses, external GRS weighting, additional stress-related loci, epigenetic markers, and postpartum follow-up are required before any precision prenatal care application can be proposed.

## Figures and Tables

**Table 1:** Baseline socio-demographic and clinical characteristics of the study cohort (*n* = 79).

Variable	Cases (*n* = 50)	Controls (*n* = 29)	Test	*p*-Value	SMD/Effect Size
Age, years, mean ± SD	29.70 ± 3.89	29.41 ± 3.40	*t* = 0.35	0.728	0.08
BMI, kg/m^2^, mean ± SD	27.01 ± 3.69	25.92 ± 2.15	*t* = 1.69	0.095	0.34
Gestational age at enrolment, weeks	24.67 ± 1.60	24.42 ± 2.18	*t* = 0.54	0.589	0.14
Higher education, *n* (%)	33 (66.0)	12 (41.4)	*χ*^2^ = 3.72	0.054	0.51
Urban residence, *n* (%)	44 (88.0)	23 (79.3)	*χ*^2^ = 0.64	0.424	0.24
Married, *n* (%)	44 (88.0)	27 (93.1)	*χ*^2^ = 0.02	0.887	−0.18
NRG1 rs35753505, CC/CT/TT	3/24/23	5/14/10	*χ*^2^ = 1.64	0.201	*V* = 0.14
NRG1 rs3924999, AA/AG/GG	6/31/13	3/14/12	*χ*^2^ = 0.78	0.376	*V* = 0.10
OXT rs2740210, AA/AC/CC	2/25/23	3/16/10	*χ*^2^ = 0.96	0.327	*V* = 0.11

**Table 2:** Antenatal psychological scores and prevalence above clinical cut-offs by group.

Instrument	Cases (*n* = 50)	Controls (*n* = 29)	*t*	*p*	Above Cut-Off, Cases vs. Controls
EPDS (0–30)	12.47 ± 4.86	7.33 ± 4.41	4.87	<0.001	28/50 (56.0%) vs. 3/29 (10.3%), *p* < 0.001
GAD-7 (0–21)	12.14 ± 4.14	6.30 ± 3.83	6.43	<0.001	38/50 (76.0%) vs. 4/29 (13.8%), *p* < 0.001
PSS-10 (0–40)	21.92 ± 5.09	16.07 ± 5.53	4.74	<0.001	37/50 (74.0%) vs. 9/29 (31.0%), *p* < 0.001
CD-RISC-25 (0–100)	54.57 ± 11.27	66.97 ± 10.20	−5.08	<0.001	34/50 (68.0%) vs. 5/29 (17.2%) <60, *p* < 0.001

**Table 3:** Hardy–Weinberg equilibrium and minor allele frequencies of the three genotyped SNPs.

SNP (Gene)	Group	Genotype Counts	MAF	HWE *χ*^2^	*p* (HWE)
rs35753505 (NRG1)	study lot	CC = 3/CT = 24/TT = 23	0.300	1.020	0.312
	Controls	CC = 5/CT = 14/TT = 10	0.414	0.001	0.979
	All	CC = 8/CT = 38/TT = 33	0.342	0.377	0.539
rs3924999 (NRG1)	study lot	AA = 6/AG = 31/GG = 13	0.430	3.506	0.061
	Controls	AA = 3/AG = 14/GG = 12	0.345	0.136	0.713
	All	AA = 9/AG = 45/GG = 25	0.399	2.791	0.095
rs2740210 (OXT)	study lot	AA = 2/AC = 25/CC = 23	0.290	2.294	0.130
	Controls	AA = 3/AC = 16/CC = 10	0.379	0.855	0.355
	All	AA = 5/AC = 41/CC = 33	0.323	2.765	0.096

**Table 4:** Exploratory carrier-based case–control analysis of the three SNPs and allele frequency comparison. This table does not represent a fully powered homozygote/heterozygote comparison within each study group.

SNP (Risk Allele)	Study Lot, Carrier/Total (%)	Controls, Carrier/Total (%)	OR (95% CI)	*p* (Fisher)	Allele *χ*^2^, *p*
NRG1 rs35753505 (T)	47/50 (94.0)	24/29 (82.8)	3.26 (0.72–14.83)	0.135	1.64, 0.201
NRG1 rs3924999 (A)	37/50 (74.0)	17/29 (58.6)	2.01 (0.76–5.31)	0.210	0.78, 0.376
OXT rs2740210 (A)	27/50 (54.0)	19/29 (65.5)	0.62 (0.24–1.59)	0.353	0.96, 0.327

**Table 5:** Pearson correlations between antenatal psychological scores and obstetric–neonatal outcomes (full sample, *n* = 79).

Outcome	EPDS	GAD-7	PSS-10	CD-RISC-25
Birth weight (g)	*r* = −0.44, *p* < 0.001	*r* = −0.55, *p* < 0.001	*r* = −0.41, *p* < 0.001	*r* = 0.49, *p* < 0.001
Gestational age at birth (wk)	*r* = −0.36, *p* = 0.001	*r* = −0.42, *p* < 0.001	*r* = −0.32, *p* = 0.004	*r* = 0.27, *p* = 0.016
Apgar at 1 min	*r* = −0.32, *p* = 0.003	*r* = −0.49, *p* < 0.001	*r* = −0.32, *p* = 0.004	*r* = 0.28, *p* = 0.011
Apgar at 5 min	*r* = −0.26, *p* = 0.017	*r* = −0.40, *p* < 0.001	*r* = −0.06, *p* = 0.578	*r* = 0.35, *p* = 0.002

**Table 6:** Multivariate logistic regression predicting NICU admission (*n* = 79 with complete genotype data).

Predictor	*Β*	*SE*	*z*	OR (95% CI)	*p*
GRS	−0.242	0.251	−0.96	0.785 (0.480–1.284)	0.335
GAD-7	0.195	0.070	2.78	1.216 (1.059–1.395)	0.006
PSS-10	0.125	0.054	2.32	1.134 (1.020–1.260)	0.020
CD-RISC-25	−0.056	0.027	−2.06	0.945 (0.896–0.997)	0.039
Maternal age	0.028	0.078	0.36	1.029 (0.883–1.198)	0.717
Intercept	−0.947	3.014	−0.31	–	0.753

Model fit: Pseudo-R^2^ = 0.30; likelihood-ratio test *p* < 0.001; log-likelihood = −38.37.

**Table 7:** Gene × Environment interaction model for low birth weight (cases only, *n* = 50). Outcome: LBW (0/1).

Predictor (Centred)	*Β*	*SE*	*z*	OR (95% CI)	*p*
GRS (mean-centred)	−0.012	0.279	−0.04	0.988 (0.571–1.708)	0.965
PSS-10 (mean-centred)	−0.005	0.063	−0.08	0.995 (0.878–1.127)	0.935
GRS × PSS-10 (interaction)	0.027	0.057	0.48	1.027 (0.919–1.148)	0.633
Maternal age	0.153	0.087	1.76	1.165 (0.983–1.381)	0.078
BMI	0.003	0.082	0.03	1.003 (0.854–1.177)	0.973
Intercept	−5.026	3.488	−1.44	–	0.150

Pseudo-R^2^ = 0.057; LR test *p* = 0.568 (underpowered multiplicative interaction model).

**Table 8:** Stratified Pearson correlations between psychological scores and birth weight within each GRS tertile (full sample, *n* = 79).

GRS Tertile (*n*)	PSS-10—BW	GAD-7—BW	EPDS—BW	CD-RISC-25—BW
Low (*n* = 31)	*r* = −0.18, *p* = 0.326	*r* = −0.25, *p* = 0.178	*r* = −0.19, *p* = 0.310	*r* = 0.33, *p* = 0.068
Mid (*n* = 24)	*r* = −0.31, *p* = 0.144	*r* = −0.47, *p* = 0.021	*r* = −0.40, *p* = 0.054	*r* = 0.42, *p* = 0.041
High (*n* = 24)	*r* = −0.58, *p* = 0.003	*r* = −0.63, *p* = 0.001	*r* = −0.55, *p* = 0.005	*r* = 0.51, *p* = 0.011

**Table 9:** Exploratory comparison of obstetric–neonatal and psychological outcomes across GRS tertiles (full sample, *n* = 79).

Outcome	GRS Low (*n* = 31)	GRS Mid (*n* = 24)	GRS High (*n* = 24)	Test	*p*
Birth weight (g)	2849.5 ± 575.9	2339.4 ± 626.8	2414.9 ± 553.8	*F* = 6.00	0.004
Apgar at 5 min	9.5 ± 0.7	9.0 ± 0.6	9.0 ± 0.6	*F* = 4.72	0.012
EPDS	8.8 ± 5.4	12.0 ± 4.2	11.8 ± 5.4	*F* = 3.27	0.043
GAD-7	8.5 ± 4.9	11.9 ± 4.7	10.5 ± 4.1	*F* = 3.74	0.028
PSS-10	16.9 ± 6.2	20.9 ± 4.6	21.9 ± 5.2	*F* = 6.27	0.003
CD-RISC-25	64.6 ± 10.1	54.5 ± 13.2	56.0 ± 10.7	*F* = 6.34	0.003
IUGR, *n* (%)	5 (16.1)	12 (50.0)	5 (20.8)	*χ*^2^ = 8.57	0.014
Gestational HTN, *n* (%)	4 (12.9)	10 (41.7)	4 (16.7)	*χ*^2^ = 7.09	0.029
Preterm birth, *n* (%)	13 (41.9)	12 (50.0)	10 (41.7)	*χ*^2^ = 0.45	0.797
Low birth weight, *n* (%)	6 (19.4)	8 (33.3)	7 (29.2)	*χ*^2^ = 1.47	0.479

## Data Availability

The data presented in this study are available on request from the corresponding author.
